# Supplementary material for: Hemin-Induced Endothelial Dysfunction and Endothelial to Mesenchymal Transition in the Pathogenesis of Pulmonary Hypertension Due to Chronic Hemolysis
Source: Int J Mol Sci. 2022 Apr 26;23(9):4763. doi: 10.3390/ijms23094763 (PMC9104708; doi:10.3390/ijms23094763)
Supplement: Supplementary file 1 [file ijms-23-04763-s001.zip › ijms-1697118-supplementary.pdf]

International Journal of
Molecular Sciences

MDPI

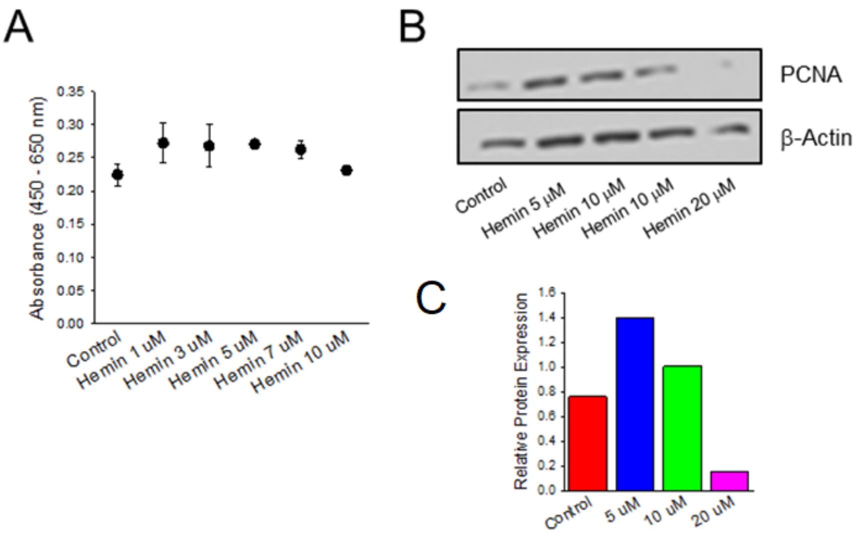

Figure S1. Hemin treatment leads to increased pulmonary artery endothelial cell proliferation at specific concentrations. (**A**) Plot depicting absorbance related to WST-1 assay as a measure of cellular proliferation in HPAECs treated for 24-hour duration with hemin at 1 µM, 3 µM, 5 µM, 7 µM, and 10 µM compared to PBS vehicle, $n = 3$. (**B**) Western blot image denoting PCNA protein expression, a marker of cell proliferation, measured between HPAECs treated with hemin 5 µM, 10 µM, and 20 µM compared to PBS vehicle for 24 hours; $n = 1$. (**C**) Bar graph summarizing relative PCNA protein expression across the experiment. PCNA indicates proliferating cell nuclear antigen.
